# The Five-Point Test: Reliability, Validity and Normative Data for Children and Adults

**DOI:** 10.1371/journal.pone.0046080

**Published:** 2012-09-24

**Authors:** Lara Tucha, Steffen Aschenbrenner, Janneke Koerts, Klaus W. Lange

**Affiliations:** 1 Department of Clinical and Developmental Neuropsychology, University of Groningen, Groningen, The Netherlands; 2 Department of Psychosomatic and General Internal Medicine, Centre for Psychosocial Medicine, University Hospital Heidelberg, Heidelberg, Germany; 3 Department of Experimental Psychology, University of Regensburg, Regensburg, Germany; University of South Florida, United States of America

## Abstract

The present study provides normative data from a sample of 257 healthy children and 608 adults on a modified version of the Five-Point Test (5PT). The 5PT is a structured and standardized test measuring figural fluency functions. Interrater reliability, test-retest-reliability and construct validity of this measure were analyzed. The sensitivity of the task for cognitive disturbances of patients with neurological diseases was proven by analyzing the test performance in the 5PT of patients with Parkinson's disease. Finally, normative data stratified by age and corrected for education level is provided. The results of the present study confirm the value of the 5PT in the measurement of figural fluency functions in clinical examination and neuropsychological research.

## Introduction

A previous study published recently in PLoS ONE [1] provided normative data of an impressive sample (n = 1651) on the Ruff Figural Fluency Test (RFFT). The RFFT is one of the most frequently used measures for the assessment of figural fluency functions which are associated with executive functioning [2]. The RFFT belongs to the more structured figural fluency tests and requires the generation of drawings of different figures (designs) in given configurations of five-dot patterns with different backgrounds. The test is characterized by a clear assessment procedure which is simple in application and scoring. However, this test has been criticized as being overstructured and therefore as being insufficiently sensitive [3]. The value of the RFFT is further limited by the 5-minute test period which requires high and constant concentration form participants. Since attention deficits however are one of the most common consequences of brain pathology [2], patients with neurological or psychiatric diseases often experience difficulties in performing the RFFT. Furthermore, patients often complain about difficulties in the identification of the dot patterns in the test conditions with distractors.

The RFFT has been developed on the basis of modifications of the Five-Point Test (5PT) as devised by Regard and colleagues [4]. The 5PT is also a structured figural fluency test requiring the generation of drawings of different figures, however in only one given configuration of symmetrically and identically arranged dots (identical to the five-dot arrangement on a dice). Therefore, this test would allow an assessment of performance in shorter test periods if normative data would be available. A recent study [5] already provided normative data of 280 adults for the 5PT which was performed for three minutes. Normative data have been stratified by age (three classes of 20 years of age each: 19–39 years, 40–59 years and 60–80 years) and education (2 classes: lower education (1–13 years of education) and higher education (above 13 years)) for the complete three-minute test period. Another recent study [6] provided cut-off scores for different test variables (e.g. number of unique designs, errors index) on the basis of 332 adults. Finally, a recent study on an Arabic sample [7] provided mean scores and standard deviations for the Arabic population on the 5PT (n = 215; age range 18–59 years). Despite these normative data and cut-off scores being helpful in the clinical setting, data from larger samples would be desirable to have narrower age groups for the assessment of performance. This is of particular importance, since as Hanks and colleagues [3] summarized, figural fluency test have been found to be sensitive to brain dysfunction even when other measures of executive functioning have been undisturbed. In addition, figural fluency measures allow the assessment of fluency functions in children [8] and patients with speech disturbances.

Since the assessment of figural fluency is important in both experimental and clinical settings and good normative data for the 5PT are scarce, this study presents normative data from a large sample of healthy children and adults on the 5PT. Previous studies already demonstrated that the 5PT can be performed in children. While the test period of the original version is five minutes [4], the present version requires the participants to generate designs for only two minutes. Normative data are given for the one-minute and the two-minute period.

## Methods

### Participants

#### Healthy adult participants

Six hundred and thirty five healthy adults all living independently in the community were included in the present study. Participants responded to public announcements or were recruited through different courses at the local university, local sports or leisure clubs, social organizations, word-of-mouth or personal contacts. They received no financial remuneration for participating in the study. A self-reported history of medical and psychiatric problems was obtained from each subject. Participants with a known history of neurological disease, psychiatric illness, head injury, stroke, substance abuse or learning disability and those taking medications known to affect the central nervous system were excluded from the study. In order to rule out gross cognitive dysfunction, in particular in elderly participants, the Mini Mental State Examination [9] was administered to all participants. This widely used examination assesses orientation, registration, attention, calculation, language and memory. Those participants with a score below 24 were excluded (N = 27). The age of the remaining 608 participants (337 female, 271 male) ranged from 20 to 88 years (M = 41.8 years, SD = 15.4 years). The education level varied from 6 to 22 years (M = 11.8 years; SD = 3.2 years). Handedness was measured using a short version of the handedness questionnaire by Rackowski, Kalat and Nebes [10], [11]. Fourty-one participants were classified as left-handed, 534 participants as right-handed and 33 participants as ambidextrous. The sample was subdivided into six age groups. The first group comprised 149 participants aged between 20 and 29 years (Table 1), the second group 141 participants aged between 30 and 39 years, the third group 136 participants aged between 40 and 49 years, the fourth group 111 participants aged between 50 and 59 years, the fifth group 40 participants aged between 60 and 69 years and the sixth group 31 participants over 69 years.

**Table 1:** Characteristics of adult groups (mean ± SD).

		Age groups					
		20–29 years (n = 149)	30–39 years (n = 141)	40–49 years (n = 136)	50–59 years (n = 111)	60–69 years (n = 40)	over 69 years (n = 31)
Gender	(female/male)	75/74	77/64	86/50	63/48	19/21	17/14
Handedness	(right-hander/ambidexter/left-hander)	122/6/21	134/4/3	119/11/6	95/9/7	37/1/2	29/2/0
IQ A		115.67±13.64	119.74±15.55	115.50±14.20	117.81±16.47	116.10±15.48	108.65±14.55
Education	(years)	12.64±1.94	12.60±3.45	11.28±3.19	11.43±3.62	10.50±3.55	9.71±3.37

A Assessment using the Multiple Choice Vocabulary Test (Lehrl et al., 1995).

#### Healthy children

In addition to adults, 257 healthy children were examined in the present study (138 female, 119 male; mean age = 11.1 years, SD = 2.4 years). Children were recruited through local schools and sports clubs, word-of-mouth or personal contacts. None of the children had any history of learning disabilities, neurological or psychiatric disease or were receiving drugs known to affect the central nervous system. Fourteen children were classified as left-handed, four as ambidextrous and 239 as right-handed. According to age, the sample of children was subdivided into four consecutive age groups (Table 2).

**Table 2:** Characteristics of children groups (mean ± SD).

		Age groups			
		8–9 years (n = 80)	10–11 years (n = 65)	12–13 years (n = 61)	14–15years (n = 51)
Gender	(girls/boys)	33/47	34/31	36/25	35/16
Handedness	(right-hander/ambidexter/ left-hander)	73/1/6	59/1/5	58/1/2	49/1/1
Education	(years)	3.00±0.90	4.71±0.74	6.80±0.81	8.53±0.81

#### Patients with Parkinson's disease

Furthermore, 15 adult patients with Parkinson's disease who had been consecutively referred to the Department of Neurology of the University of Würzburg (Germany) as outpatients participated in the present study. Parkinson's disease was diagnosed according to the UK brain bank criteria [12]. Patients with Parkinson's disease were assessed on their usual antiparkinson medication. According to Hoehn and Yahr [13], severity of clinical symptoms of patients with Parkinson's disease was rated as Stage II (4 patients), Stage III (9 patients) or Stage IV (2 patients). Characteristics of patients are presented in Table 3.

**Table 3:** Characteristics of patients with Parkinson's disease and healthy participants (mean ± SD).

	Healthy participants (n = 15)	Patients with PD (n = 15)	t	P
Gender (female/male)	9/6	9/6		
Age (years)	64.33±8.78	64.04±8.23	1.05	.313
IQ	109.00±14.66	104.93±15.10	1.69	.112
Education (years)	9.20±2.68	8.73±1.87	1.24	.235

PD  =  Parkinson's disease.

### Ethics statement

This study was approved by the ethics committees of the University of Regensburg, Germany, and University of Würzburg, Germany. Prior to inclusion in the study, all adult participants signed an informed consent and parents gave written informed consent for their children to participate in the study. All patients with Parkinson's disease gave written informed consent to take part in the study. Each patient's capacity to consent was evaluated by a consultant neurologist. Only patients without dementia were included in the study (i.e. patients scoring higher or equal 26 in the Mini Mental State Examination).

### Design and Procedure

All participants completed a modified version of the Five-Point Test (5PT). The only modification of the test applied in the present study was a reduction of the test period: The modified version was performed for two minutes, while the original version of the 5PT as devised by Regard and colleagues [4] was performed for three minutes. Furthermore, intellectual function (IQ) was measured in all adult participants using the Multiple Choice Vocabulary Test [14], a valid and short test procedure for the estimation of intelligence on the basis of word knowledge. Furthermore, 75 adult participants of the sample (37 female, 38 male; mean age  = 40.3 years, SD  = 12.5 years) completed both a letter and a semantic fluency task, the Digit Span Forward and Backward Tasks of the Wechsler-Memory-Scale-Revised (WMS-R; Wechsler, 1987), the Visual Memory Forward and Backward Tasks (WMS-R), the Logical memory I and II (WMS-R), the copy and recall administration of the Complex Figure Test [15], [16], the Stroop Color and Word Test [17], the Tower of London task [18] and both parts of the Trail-Making Test [19]. Participants were recruited as outlined above (see section on ‘Healthy adult participants’). For the analysis of test-retest-reliability, a sample of 50 students (25 female, 25 male; mean age  = 21.78 years, SD  = 2.67 years) was asked to complete the 5PT twice. These participants were recruited at the University of Regensburg (Germany) via poster advertisements and word of mouth. The time period between the first and second testing was three weeks.

### Materials

In the *5PT* [4] the participant was presented with sheets of paper on which 40 squares, each consisting of a fixed pattern of five symmetrically arranged dots, were printed. The participant was asked to produce as many different designs as possible by connecting the dots in each square with one or more straight lines within two minutes. The participant was given the following instructions:

*“There are a number of squares, each containing five dots, on the sheet of paper in front of you. I want you to draw as many designs as possible within the next two minutes by connecting two or more dots with straight lines. Not all dots per square have to be used. Please do not repeat any designs or draw lines that do not connect dots.”*

After the demonstration of two possible designs by the examiner, the participant was asked to begin with the task. Repetitions of designs (perseverative errors) and production of designs with lines that failed to connect dots (rule violations) were regarded as errors. The number of unique designs and the number of both perseverative errors and rule violations were scored for each minute.

In the *letter fluency task* (M-Word Test), the participants were given two minutes to produce as many different words as possible beginning with the letter “m”. Names (e.g., Mike, Michigan, Mexico), words with the same stem (e.g., master, master copy, master key), words beginning with another letter, non-existent or foreign-language expressions (rule violations) as well as repetitions (perseverative errors) were classed as errors.

In the *semantic fluency task* (Animal Naming), the participants were asked to name as many animals as possible within two minutes. Similarly, deviations from test rules were classed as errors, including perseverative errors and words which were not identifiable as animal names (rule violations). All words named by the participant were recorded by the examiner.

In the *Digit Span Forward* and *Digit Span Backward Tasks* [20], a series of digits is read to the participant who is required to repeat the digits either in the order given or in the reverse order. These tests measure short-term memory and working memory, respectively, for verbal stimuli.

In the *Visual Memory Span Forward* and *Backward Tasks* [20], the examiner demonstrates a series of tapping sequences on a pattern consisting of eight rectangles. The participant is requested to repeat these series either in the order given or in the reverse order. These tests measure short-term memory and working memory, respectively, for figural stimuli.

The *Logical Memory I* and *II* [20] requires the participant to listen to two short stories and to recall as many details as possible from each story immediately and after approximately 30 minutes. These tests measure immediate and delayed recall of verbal information.

In the *Complex Figure Test* devised by Rey [16] and Osterrieth [15], the participant is asked to copy a geometric figure (copy administration). Without forewarning, the participant is asked after a time delay of 30 minutes to recall the complex figure already drawn on the copy administration. Criteria of accuracy scoring are published by Lezak [2]. This test measures visuoconstructive abilities and delayed recall of figural information.

In the *Stroop Test* [17] three cards are shown to the subject. On the first card (color word subtest) the subject is asked to read a list of color words printed in black ink (yellow, blue, green and red). On the second card (color name subtest) the subject is requested to name the color of rectangles. On the last card (interference subtest) a list of color words printed in a different color ink (e.g. the word red is printed in blue) is used. The subject has to name the color and ignore the written word. Time is measured in all three conditions. In the last part of the test (interference subtest) both the number of mistakes and the number of self-corrections are counted. This test measures inhibition.

The *Tower of London* task *(TOL)* consists of a board with three vertical pegs of different size on which three beads of different colors can be arranged [18]. The task requires the participant to move the beads from a starting position to a target position, which is illustrated on a card, using the minimal number of moves which is specified by the examiner. The task used in the present study consists of 20 problems ranging in level of difficulty from three to six moves. There were five problems in each level of difficulty. The rules specify that only one bead may be moved at a time and that once a bead is picked up it must be placed on a peg. Time for planning and the number of problems solved in the minimal number of moves are recorded. Planning time was operationalized as the time period between the presentation of a card and the beginning of the participant's movement. The Tower of London task measures planning and problem solving abilities.

The *Trail-Making Test, Part A* and *B* [19] requires participants to connect a series of digits placed in random order on a sheet of paper in ascending order and to connect a series of numbers and letters in ascending order alternating between numbers and letters (i.e. 1-A-2-B-etc.). These tests measure processing speed and mental flexibility, respectively.

### Data Analysis

Product moment correlations of Pearson were calculated between both age and years of education and the number of unique productions, perseverative errors and rule violations made by participants within a test period of two minutes during the 5PT. These associations were computed in order to find out whether figural fluency performance is influenced by age and education. Since age of children corresponds with years of education, correlation analysis between the 5PT and years of education was performed only for adults. Comparison between the performance of males and females regarding figural fluency was performed using analysis of covariance.

Interrater-reliability was analyzed by correlating the scorings of two independent novice raters who were asked to evaluate the test protocols of 60 adult participants (30 female, 30 male; mean age  = 45.8 years, SD  = 9.3 years) who had performed the 5PT (test period of two minutes). This subgroup of 60 participants was selected at random from the whole sample of 608 adult participants. Intraclass correlation coefficients (ICC), as devised by Shrout and Fleiss [21], were calculated as measures of interrater agreement (ICC, 2.1) and rater consistency (ICC, 3.1) [22]–[24]. In contrast to the Pearson product-moment correlations, these intraclass correlations consider the variance-covariance matrices of raters' ratings. Test-retest-reliability was analyzed according to the suggestions of Lord and Novick [25]. Furthermore, participants' performance (N = 50) on the initial testing was compared with their test results on the second testing using t-tests for paired samples.

For the analysis of construct validity, product moment correlations of Pearson were calculated between the number of unique designs, perseverative errors and the number of rule violations performed by adult participants in the 5PT within a test period of two minutes, and their performance regarding word fluency, short-term and working memory, immediate and delayed recall of information, visuoconstructive abilities, inhibition, problem solving, processing speed, mental flexibility and intellectual functioning (IQ). This broad variety of functions was assessed, since the evaluation of construct validity requires that various measures of the same construct are strongly associated to each other (convergent validity) and less strongly to measures of other constructs (discriminant validity).

Sensitivity of the 5PT for cognitive disturbances of patients with neurological diseases was assessed by comparing the test performance of adult patients with Parkinson's disease with the test results of healthy adult participants using t-test for paired samples. Patients were matched to healthy participants according to sex, age, handedness, education and intellectual functions (Table 3). All patients and controls were right-handed.

For statistical analysis an alpha level of .05 was applied. All statistical analyses were carried out using the Statistical Package for Social Sciences 16.0 for Windows.

## Results

### Demographic characteristics

Correlation analysis between demographic characteristics of adult participants revealed a significant relationship between age and both the number of unique designs (N = 608, r = −.619, p<.001) and the number of rule violations (N = 608, r = .084, p = .038) within a test period of two minutes. The number of perseverative errors was not related to age (N = 608, r = .014, p = .737). Similar results were found regarding years of education. While the number of unique designs (N = 608, r = .346, p<.001) and the number of rule violations (N = 608, r = −.106, p = .009) within a test period of two minutes were significantly correlated with years of education, the number of perseverative errors was not related to education (N = 608, r =  −.048, p = .237). According to Cohen [26], the correlation between age and the number of unique designs was large (r>.50). The relationship between the number of unique designs and years of education was medium (r>.30). The remaining relationships were small (r>.10) or of negligible magnitude (r<.10). Probably, the negligible to small correlations only reached significance as a result of the large sample size because sample size and power of a statistical test are closely related [26].

Comparison of demographic variables between healthy women and men using t-tests for independent samples revealed a significant difference in years of education (women: mean education  = 11.3 years, SD  = 2.9 years; men: mean education  = 12.5 years, SD  = 3.5 years; t = −4.37; p<.001) but not in age (women: mean age  = 42.0 years, SD  = 15.2 years; men: mean age  = 41.4 years, SD  = 15.8 years; t = 0.47 p = .641). Statistical comparisons between healthy women and men were therefore made using analysis of covariance. Since figural fluency functions, as assessed by the 5PT, have been shown to be related to education, years of education was used as covariate. Analysis of covariance revealed no significant differences between women and men with regard to the number of unique designs and the number of rule violations in the 5PT. However, women produced significantly more perseverative errors than men (Table 4). The analysis of effect sizes for group differences revealed only small (d<0.5) to negligible effects (d<0.2).

**Table 4:** Performance of healthy adults and children on the Five-Point Test (mean ± SD).

Healthy adult participants					
	Women (n = 337)	Men (n = 271)	F	P	Effect size
Correct productions	27.94±9.35	29.86±9.33	1.14	.286	0.21
Perseverative errors	1.95±2.88	1.34±1.94	0.82	.004 **	0.25
Rule violations	0.07±0.38	0.02±0.19	1.98	.160	0.17
Healthy children					
	Girls (n = 138)	Boys (n = 119)	F	P	Effect size
Correct productions	20.73±8.64	18.84±9.07	0.00	.984	0.21
Perseverative errors	0.62±0.95	0.80±1.20	2.13	.146	0.17
Rule violations	0.17±0.56	0.16±0.50	0.23	.634	0.02

** p≤.01 (analysis of covariance).

Due to the significance of age and level of education on figural fluency functions in adults, normative data for the 5PT are reported stratified for age (Table 5). According to age the sample was subdivided into six consecutive age groups (Table 1). Analogous to the procedure used in the Ruff Figural Fluency Test [27] a correction was calculated for three levels of education (under 10 years, 10–12 years and over 12 years of education). This educational correction represents the differences between cell means (stratified for age and education) and the mean of the complete sample. In order to obtain an education corrected normative value of an individual, the corresponding score on Table 6 must be added to the individual raw score (percentiles in Table 5).

**Table 5:** Percentiles for adults for the Five-Point Test.

	One – minute period						Two – minute period						
Raw Score													Raw Score
	20–29 years	30–39 years	40–49 years	50–59 years	60–69 years	over 69 years	20–29 years	30–39 years	40–49 years	50–59 years	60–69 years	over 69 years	
3				1		6						3	3
4				3	2	19							4
5	1			5	7	42						10	5
6	2			6	12	52				1		13	6
7			2	7		71						16	7
8	3	1	4	10		84				3		26	8
9	3		5	12	20	90				5	5	39	9
10	4	3	8	16	35	94				6	12	48	10
11	6	4	9	23	42			1	1	7	15	55	11
12	9	6	14	27	57	97					17	61	12
13		8	17	35	65	99			2	8		71	13
14	10	10	23	41					3		20	81	14
15	13	14	36	51	70		1			12	25	84	15
16	16	19	45	62	78		2		4	13	35	90	16
17	19	24	52	69	85					15	40	97	17
18	24	32	61	71	90				7	21	45		18
19	33	41	70	80	95		5		11	22	50		19
20	39	53	78	83			6	2	14	27	55		20
21	48	61	82	88			7	6	16	31	57		21
22	56	70	85	89			8	8	18	33	60		22
23	64	72	90	92				13	23	41	62	99	23
24	74	78	92	94			9	15	32	50	68		24
25	81	85	96	98			11	16	38	56	72		25
26	84	89	98	99	98		13	23	46	60	82		26
27	88	93	99		99		15	30	49	60			27
28	90	94					18	32	51	62	85		28
29	92						20	38	57	68	87		29
30	94	96					25	43	62	74	90		30
31	95	98					26	48	70	76			31
32	98	99					30	54	75	81			32
33	99						40	61	78	86	95		33
34							46	63	81	88			34
35							58	70	87	91			35
36							65	76	88	95	98		36
37							70	81		96			37
38							72	83	92	97			38
39							79	86	93	98			39
40								89	95				40
41							82		96				41
42							83	92	98				42
43							85	94	99	99			43
44							89	96					44
45							90						45
46							95	98					46
47							97						47
48													48
49													49
50								99					50
51							98						51
52							99						52

**Table 6:** Correction of raw scores for three education levels.

	One – minute period						Two – minute period						
Years of education													Years of education
	20–29 years	30–39 years	40–49 years	50–59 years	60–69 years	over 69 years	20–29 years	30–39 years	40–49 years	50–59 years	60–69 years	over 69 years	
Under 10	+5	+3	+2	+2	+1	0	+4	+5	+4	+3	+2	0	Under 10
10–12	+2	+1	0	0	−2	0	+3	+2	0	0	−3	0	10–12
Over 12	−1	−2	−2	−2	−2	−1	−1	−2	−3	−3	−3	−3	Over 12

For example: A 45-year old subject with nine years of education generated 22 different designs during the two-minute test period of the 5PT (raw score  = 22). This performance corresponds to a percentile of 18 (Table 5). In order to consider the level of education (9 years), a value of four must be added to the original score of 22 (Table 6). The education corrected percentile can then be taken from Table 5 by using the new raw score of 26. This education corrected raw score corresponds to a percentile of 46.

Correlation analysis between years of age and figural fluency performance of children displayed a significant relationship between age and the number of unique designs (r = .548, p<.001). The remaining correlation coefficients were of small (rule violations: r = −.115, p = .066) or negligible size (perseverative errors: r = .025, p = .692). Comparison of demographic variables between healthy girls and boys using t-tests for independent samples revealed a significant difference in both age (girls: mean age  = 11.5 years, SD  = 2.3 years; boys: mean age  = 10.6 years, SD  = 2.4 years; t = 3.20; p = .002) and years of education (girls: mean education  = 5.8 years, SD  = 2.2 years; boys: mean education  = 5.0 years, SD  = 2.2 years; t = 2.91; p = .004). Statistical comparisons between girls and boys were therefore made using analysis of covariance. Since age of children corresponds with years of education (r = .946; p<.001) only years of age was used as covariate. Analysis of covariance revealed no significant differences between girls and boys in any of the measures of the 5PT (Table 4). The analysis of effect sizes for group differences revealed only small (d<0.5) to negligible effects (d<0.2). Normative data for children groups are presented in Table 7.

**Table 7:** Percentiles for children for the Five-Point Test.

	One – minute period				Two – minute period				
Raw Score									Raw Score
	8–9 years	10–11 years	12–13 years	14–15 years	8–9 years	10–11 years	12–13 years	14–15 years	
3	9	1							3
4	26		2						4
5	36	5	10						5
6	49	9	13	2	10				6
7	54	21	16	6	19	2	3		7
8	64	29	23	12	26	3	5		8
9	69	35	25	18	36	8	10		9
10	74	45	38	20	40	12			10
11	82	52	41	24	44	14		4	11
12	90	55	46	26	49	21	13	6	12
13	93	57	51	35	55	23	15	8	13
14	94	66	61	39	64		20	10	14
15	96	71	67	45	71	29	23		15
16		78	72		75	37		12	16
17		83	75	53	78	43	25	18	17
18		86	77	57	79	46	30		18
19		89	82	61	83	49	36	24	19
20		95	84	69		51	38	26	20
21		98	92	71	88	57	50	28	21
22			93	73	90	62	53	31	22
23			95	84	94	70	61	37	23
24				90		72		39	24
25				92	96	75	66		25
26				98			67	43	26
27							72	51	27
28						79	77		28
29						85	85	57	29
30						91		63	30
31							88	69	31
32						92	90	73	32
33						95		80	33
34								86	34
35						97		88	35
36						99	93		36
37								94	37
38							98		38
39									39
40								98	40

### Interrater reliability

The simple scoring criteria of the 5PT resulted in an excellent rater agreement (ICC, 2.1) with nearly perfect to perfect indices (unique desgins: r = 0.999; preservative errors: r = 0.998; rule violations: r = 1.000). The coefficients for the rater consistency (ICC, 3.1) were the same as the coefficients for the rater agreement, indicating a high interrater reliability.

### Test-retest reliability

Intraclass correlation analysis [21] revealed a correlation coefficient of r = .65 between the two sets of scores for the number of unique designs generated within one minute. Regarding the number of unique designs produced within the two-minute test period the test-retest reliability was acceptable (r = .77). However, the test performance of participants' regarding the number of unique designs improved significantly from initial testing to retesting (Table 8), indicating medium differences (d>0.5). This improvement by about four unique designs may indicate a change with practice. The low level of perseverative errors and rule violations did not necessitate an analysis of test-retest-reliability of errors. However, the number of both perseverative errors and rule violations did not differ between the initial testing and the retesting. Furthermore, these differences represent negligible effects (d<0.2).

**Table 8:** Test scores of 50 students in the Five-Point Test on the initial testing and the retesting (mean ± SD).

	Initial testing	Retesting	t	p	Effect size
Test performance for the one-minute test-period					
Correct productions	23.87±4.88	27.21±4.47	−4.82	<.001 **	0.71
Perseverative errors	0.77±1.29	0.62±1.01	0.81	.425	0.13
Rule violations	0.00±0.00	0.14±0.88	−1.15	.254	0.16
Test performance for the two-minute test-period					
Correct productions	37.59±6.43	42.08±7.57	−5.14	<.001 **	0.63
Perseverative errors	1.76±2.15	1.80±2.08	−.10	.923	0.02
Rule violations	0.00±0.00	0.14±0.88	−1.15	.254	0.16

** p≤.01 (t-test for paired samples).

### Construct validity

According to Cohen [26], the correlations between the number of unique designs and processing speed, mental flexibility and both the number of unique productions in both the letter fluency task and the semantic fluency task were large (r≥.50). The relationships between the number of unique designs and figural short-term memory, figural working memory, problem solving and inhibition were medium (r≥.30). The size of the remaining correlations were small or negligible (r<.30). However, the relationship between the number of unique designs in the 5PT and the number of perseverative errors in the semantic fluency task, the measures of verbal memory, the delayed recall for figural information and visuoconstructive abilities reached significance. Furthermore, a significant association between the number of rule violations in the 5PT and both visuoconstructive abilities and mental flexibility was found. Table 9 provides product moment correlations of Pearson for measures of cognitive functioning, with the number of unique designs, perseverative errors and violations made by healthy adult participants within a test period of two minutes in the 5PT.

**Table 9:** Product moment correlations of the Five-Point Test with other measures of cognitive functioning.

Cognitive function	Test procedure	Five-Point Test		
		Correct productions	Perseverative errors	Rule violations
Intellectual functions	Multiple Choice Vocabulary Test	r = .164; p = .159	r = −.177; p = .130	r = −.187; p = .108
Letter Fluency	M-Word Test Correct productions Perseverative errors Rule violations	r = .545; p<.001 ** r = −.014; p = .903 r = −.051; p = .666	r = −−.215; p = .064 r = −.034; p = .772 r = .170; p = .144	r = −.205; p = .078 r = −.089; p = .445 r = −.019; p = .870
Semantic Fluency	Animal Naming Correct productions Perseverative errors Rule violations	r = .645; p<.001 ** r = −.280; p = .015 * r = .164; p = .160	r = −.118; p = .314 r = −.202; p = .081 r = −.016; p = .890	r = −.183; p = .115 r = −.030; p = .800 r = −.014; p = .908
Verbal Memory Working memory Short-term memory Immediate recall Delayed recall	Digit Span Backward Digit Span Forward Logical memory I Logical memory II	r = .286; p = .013 * r = .238; p = .040 * r = .254; p = .028 * r = .251; p = .030 *	r = −.175; p = .134 r = −.177; p = .317 r = −.145; p = .215 r = −.179; p = .124	r = −.180; p = .123 r = −.118; p = .312 r = −.085; p = .469 r = −.139; p = .235
Figural Memory Working memory Short-term memory Delayed recall	Visual Memory Span Backward Visual Memory Span Forward Complex Figure Test (recall)	r = .333; p = .003 * r = .349; p = .002 * r = .275; p = .017 *	r = −.157; p = .180 r = −.178; p = .128 r = −.108; p = .355	r = −.087; p = .458 r = −.059; p = .617 r = −.093; p = .426
Visuoconstructive abilities	Complex Figure Test (copy)	r = .241; p = .037 *	r = −.103; p = .377	r = −.249; p = .031 *
Inhibition	Stroop Color and Word Test Reading of color words Color naming Interference condition	r = −.474, p<.001 ** r = −.313, p = .006 ** r = −.316, p = .006 **	r = −.026, p = .825 r = −.019, p = .868 r = −.108, p = .357	r = .033, p = .640 r = .186, p = .110 r = .013, p = .911
Problem solving	Tower of London	r = .321, p = .005 **	r = −.162, p = .158	r = −.066, p = .576
Processing speed	Trail-Making Test, Part A	r = −.516, p<.001 **	r = −.034, p = .773	r = −.080, p = .469
Mental flexibility	Trail-Making Test, Part B	r = −.504, p<.001 **	r = .105, p = .371	r = .279, p = .015 *

** p≤.01; * p≤.05.

### Sensitivity

Comparison of figural fluency functions between patients with Parkinson's disease and healthy participants revealed marked impairments (Table 10). Patients with Parkinson's disease produced significantly fewer unique designs than healthy participants. These differences could be observed in both the one-minute- and the two-minute test period. Patients did not differ from healthy participants in regard to the number of perseverative errors or rule violations. The effect sizes for group differences between healthy participants and patients with Parkinson's disease in regard to the number of unique designs were large (d>0.8). In contrast, the effect sizes for differences concerning both the number of perseverative errors and rule violations in the 5PT were small (d<0.5) or negligible (d<0.2).

**Table 10:** Performance of patients with Parkinson's disease and healthy participants in the Five-Point Test (mean ± SD).

Five-Point Test	One-minute test-period					Two-minute test-period				
	Healthy participants	Patients with PD	t	p	Effect size	Healthy participants	Patients with PD	t	p	Effect size
Correct productions	11.60 ±4.22	8.07 ±1.87	2.86	.013 *	0.80	20.67 ±8.64	13.27 ±2.99	3.20	.006 **	0.88
Perseverative errors	0.60 ±0.99	0.53 ±1.30	0.15	.885	0.05	1.67 ±1.76	2.33 ±5.08	−0.44	.669	0.32
Rule Violations	0.00 ±0.00	0.07 ±0.26	−1.00	.334	0.27	0.00 ±0.00	0.07 ±0.26	−1.00	.334	0.27

PD  =  Parkinson's disease; ** p≤.01; * p≤.05 (t-test for paired samples).

## Discussion

Impairments of figural fluency have been found in individuals with various neurological or psychiatric conditions including attention deficit hyperactivity disorder, dementia, dyslexia, epilepsy, fetal alcohol syndrome, intracranial mass lesion, nonfunctioning pituitary macroadenoma, personality disorder, major depression, obsessive-compulsive disorder, polysubstance abuse, stroke and traumatic brain injury [28]–[41]. Consequently, the measurement of figural fluency functions became an inherent part of neuropsychological assessment as performed in clinical settings but also of assessments performed in the context of research (e.g. [42]–[43]. However, a reasonable application of neuropsychological test procedures requires the availability of normative data. While normative data have been available for the RFFT from the normative studies of Ruff and colleagues [42], [44] which have recently been significantly extended by Izaks and colleagues [1], normative data for the 5PT were not available until recently.

During the last three years, three studies published reference data for the 5PT [5]–[7]. These data, however, were based on small sample sizes (215, 280 or 332 participants respectively) with some samples being considerably restricted in age (e.g. 18–59 years or 16–60 years). Consequently, stratification of normative data with regard to age (and education) becomes very difficult. This might be the reason why some studies reported regression analysis or mean scores and standard deviations instead of normative data stratified by age. Since figural fluency performance has reliably been shown to be associated with age and education (e.g. [1], [3], [5], [6], [42]), these variables appear to be of particular importance when providing reference data for figural fluency tests. Another restriction of available reference data for the 5PT is that one of the three studies [7] provided data of an Arabic sample which made these data very valuable for the assessment of people with an Arabic background. Because of the different writing orientation of Arabic script (from right-to-left), however, these data are difficult to apply to people with a non-Arabic background, in particular since writing direction has been shown to affect performance on other spatial tasks [45].

In general, reference data from larger samples are desirable, since they are more reliable and allow better stratification (e.g. by age). Therefore, the RFFT in combination with the reference data provided by Izaks and colleagues [1] should be the measure of choice when assessing figural fluency. The RFFT is an expanded version of the 5PT consisting of five conditions differing from one another in regard to the stimuli presentation. Due to the long test period of five minutes and the complexity of the stimuli patterns, the RFFT cannot always be performed in the examination of children, elderly subjects and patients with brain damage. Since no differences were found between the five parts of the test (Ruff, 1988), only reference data for the total scores are provided so far. Consequently, available reference data cannot be used unless the participant completes the entire test procedure.

To provide researchers and clinicians with reference data allowing the assessment and evaluation of figural fluency functions for shorter test periods, the present study presents normative data of 608 healthy adult participants for the 5PT. Since figural fluency measures can easily be used in the examination of children [4], [44], [46], [47], reference data of 257 healthy children are also presented. The 5PT is a well structured test procedure which possesses solid psychometric test properties. The standardization of the application of the test procedure and the clear instructions for the evaluation of test performances ensure a high level of objectivity. The analysis of the interrater reliability of two independent raters revealed nearly perfect to perfect interrater agreement and rater consistency. Furthermore, for the number of unique designs within two minutes, an acceptable test-retest reliability for a time period of three weeks could be shown. This finding corresponds with the results of Fernandez and colleagues [48] who also found an acceptable test-retest reliability for a period of about five weeks (on average). Furthermore, an acceptable internal consistency (split-half reliability) of the 5PT has been reported with regard to the number of unique designs [48]. The strength of these relations confirms the stability of fluency performance over time as shown in measures of verbal fluency [2], [49]. However, comparison between participants' figural fluency functions on the initial testing and their results on the second testing revealed a significant improvement with practice from the first to the second testing. On the second testing, participants produced more unique designs than during the first examination. This confirms the finding of Goebel and colleagues [5] who also found performance improvements of their participants (N = 34) after a period of four weeks with regard to the number of unique designs and the percentage of perseverations. The improvement found in the present study can be seen during the first minute of the task and remains stable during the second minute. In addition, test-retest-reliability regarding participants' test performance during the first minute of the 5PT was lower than for the two-minute test period. A significant improvement over time of figural fluency functions was also found in children who were tested twice within a period of two to three weeks [44], [47]. On the basis of the findings of DesRosiers and Kavenagh [49] who found, following repeated administration of a formal verbal fluency task, a significant improvement with practice in participants without brain damage but not in patients with closed head injury, one can assume that the test-retest-reliability is higher in patients with brain damage than in the present sample of students who were informed about the second testing in advance.

The analysis of the construct validity revealed that the 5PT is closely related to other measures of cognitive functioning, including both a letter and a semantic verbal fluency task, the Visual Memory Span Tasks of the WMS-R, the Tower of London Test, the Stroop Color and Word Test and both parts of the Trail-Making Test. The close relationships between the 5PT and both the Trail-Making Test and the Stroop Color and Word Test could be explained by the fact that all these tests procedures are timed tasks. This assumption is supported by the finding of Evans and colleagues [47] who found that motor performance, as operationalized by finger-tapping speed, influences the test performance of children on the Ruff Figural Fluency Test. The medium to large correlations between the 5PT and both the verbal fluency tasks and the Tower of London Test indicate a high convergent validity. These results provide some evidence that all these tasks measure aspects of the same construct [50], e.g. processes of thinking or, when considering the relations between the 5PT and the Visual Memory Span Tasks or the Stroop Test, executive functioning. The non-significant correlations between the 5PT and measures of verbal memory, intellectual functioning and visuoconstructive abilities can be seen as a sign of acceptable discriminant validity.

It is well known that normal aging, sex and education affects cognitive functioning [2]. A number of studies have been performed in order to assess the influence of these demographic characteristics on verbal fluency. Although age and sex differences in verbal fluency functions have not been found consistently, the majority of studies found younger adults to perform better than older adults [51]–[53]. Furthermore, women seem to have an advantage in verbal fluency tasks [53]. In contrast to these findings, the effect of education and intelligence on verbal fluency has been reliably demonstrated. Higher levels of education or intelligence have been shown to be associated with better performance [52], [54]. Several studies found both age and education related differences in figural fluency functions of adults [1], [3], [5], [6], [42]. In these studies, younger subjects and subjects with higher levels of education performed better than older subjects and subjects with lower levels of education, respectively. No differences were observed between women and men. In accordance with the literature, the present study revealed that increased age results in decreased productivity in the 5PT. Furthermore, figural fluency functions were closely related to education but not to sex. In addition, our results confirm the findings of former studies [4], [44] which demonstrated that figural fluency functions in children are age- but not sex-dependent. Since figural fluency functions of healthy adults, as assessed with the 5PT, have been shown to be sensitive to the effects of age and education, normative data for adults are presented for six age levels (Table 5). Furthermore, a correction for education level was calculated (Table 6). Normative data for children are presented for four age levels (Table 7).

In the present study, the 5PT was performed for two minutes and normative data are presented for the one-minute and the two-minute period. These two test periods are suitable for the assessment of children and elderly subjects and, in particular, for the assessment of patients with dementia or patients with brain pathologies involving an increased fatigue. The present study demonstrated that figural fluency functions were markedly reduced in patients with Parkinson's disease as compared to healthy participants. These differences could be observed after the two-minute test period but were also already obvious after the first minute of testing. This shows the 5PT to be a sensitive measure to the effects of brain dysfunction. Although normative data are presented for both the one-minute- and the two-minute period, we recommend a two-minute test period, since a study assessing verbal fluency functions revealed no differences between healthy subjects and patients with brain damage within the first 30-second period. After the first 30-second period, the productivity of patients with brain damage decreased rapidly and remained low for the rest of the allocated production time [55]. A test period of two minutes would, therefore, be more sensitive in detecting cognitive impairment in patients with mild brain damage. Where the test procedure cannot be performed for two minutes, e.g. due to reduced attentional capacities or time constraints, the 5PT can be stopped after one minute. This provides flexibility for the examiner in the application of the 5PT, in particular during assessments in the clinical context.

This study provides percentiles of test performance only for the number of unique designs. Perseverative errors and rule violations were found to be rare in healthy participants but also patients with neurological or psychiatric diseases (e.g. [56]). Since the reliability of examinations based on the observation of rare and random events is usually low, percentiles of the number of perseverative errors and rule violations were not calculated. However, in the individual assessment of a patient's cognitive abilities the quality of errors should be considered. The closer examination of error types in patients with brain damage may give information about their test comprehension or the existence of behavior problems such as perseverative behavior [2].

The present results must be viewed in the context of some limitations. First, since participants did not receive any financial remuneration for participation, it has to be assumed that those participants taking part on more extended assessments (e.g. examination of the test-retest-reliability of the 5PT which made participation on two assessments in a period of three weeks necessary) might be more motivated and interested in taking part on cognitive assessments than other individuals of the general population. Therefore, the generalization of the present findings with regard to the test-retest-reliability and possibly also the construct validity might be limited. Another limitation is that the information of participants' history of medical and psychiatric problems based solely on participants' self-report instead of a full neurological and psychiatric examination. Self-report measures are criticized for low reliability and therefore, our sample might not be totally free of participants with a neurological or psychiatric condition. Since these conditions usually adversely affect cognitive functioning, the present normative data are more conservative in case that participants with neurological or psychiatric conditions have been included; i.e. when an individual performs within the impaired range on the basis of the presented data, the participant would also perform in the impaired range if participants with neurological or psychiatric conditions would have been excluded (e.g. on the basis of a full neurological and psychiatric examination). With regard to the calculation and discussion of the construct validity of the measure presented in this study, it would have been desirable to include the original version of the 5PT or the RFFT which has not been done in this study to avoid learning or carry-over effects. In the present study, we performed the 5PT only on a limited sample of 15 patients with Parkinsons' disease who were without dementia and on medication at the time of assessment. This represents a highly selected group. Consequently, the generalizability of the present results (i.e. sensitivity of the measure) to other neurological diseases is restricted. A final limitation is that intellectual functioning (IQ) of adult participants was estimated by using a short test procedure (because of time constraints) instead of performing a full scale intelligence test. Even though the measure used in this study has been shown to be valid, it tends to overestimate intellectual functioning relative to full scale intelligence tests.

In summary, normative data for a modified version of the 5PT, a measure for figural fluency functions, is provided. The present study and previous research demonstrated that the test procedure possesses solid psychometric test properties and is easy to perform in children, elderly subjects and patients with neurological conditions, such as Parkinson's disease. Given the necessity of executive functions in everyday functioning and the frequency of impairments of these functions after brain lesion, the accurate quantification of test performance is of considerable importance. The normative data presented in this study may therefore be helpful in both the research and applied setting, in particular in the assessment of individuals with limited attentional capacities.
